# Relation between parent and child or peer alienation and life satisfaction: The mediation roles of mental resilience and self-concept clarity

**DOI:** 10.3389/fpsyg.2022.1023133

**Published:** 2022-11-17

**Authors:** Na Chen, Yuanluo Jing, Yazhi Pang

**Affiliations:** ^1^School of Psychology, Southwest University, Chongqing, China; ^2^School of Agronomy and Biotechnology, Southwest University, Chongqing, China

**Keywords:** parent-child or peer alienation, mental resilience, self-concept clarity, life satisfaction, relation

## Abstract

The research on life satisfaction originated in the 1960s and has not been completely studied yet. Life satisfaction is an index related to the state and quality of individual life. With the development of society, the relevant variables affecting life satisfaction have also changed with the times. The purpose of this study is to research the relationship between parent and child or peer alienation, mental resilience, self-concept clarity and life satisfaction, finding the mechanism of action among parent-child or peer alienation, mental resilience, self-concept clarity, and life satisfaction. This cross-sectional study recruites randomly 1,347 adolescents from six middle schools in Chongqing, China, participating in a questionnaire on sociodemographic characteristics, the Inventor of Parent and Peer Attachment (IPPA), the Connor-Davidson Resilience Scale (CD-RISC-10), the Self-concept Clarity Scale (SCCS), and the Satisfaction with Life Scale (SWLS). The sample consists of 62.4% female participants (*n* = 841) and 37.6% male participants (*n* = 506) aged from 11 to 17 years old (Mean = 14.54, SD = 1.21). We use SPSS 26 to perform the statistical analysis. The study finds that mental resilience—self-concept clarity have mediating effect on the parent or peer alienation to life satisfaction, to the effect that, parent-child or peer alienation explain life satisfaction through the chain mediating effect of mental resilience—self-concept clarity. This study explores the negative multi-use of parent-child or peer alienation on life satisfaction and provides a new perspective for the improvement of life satisfaction of adolescents.

## Introduction

Life satisfaction is defined as the assessment of life quality according to individual themselves (Shin and Johnson, 1978). Life satisfaction is also an essential concept in the field of positive psychology, which centers around mental wellbeing and a series of positive individual, behavioral, psychological outcome (Diener and Diener, 2009). Life satisfaction is also an essential factor for the growth of the teenagers that would influence their health, emotions, and so on. Life satisfaction is related to the cognitive component of wellbeing (Xiang et al., 2020), and is the subjective assessment of people’s lives (Brannan et al., 2013). It is acknowledged that when people have positive emotions, they will be happier and feel a sense of wellbeing more strongly than people who have less positive emotions, thus improve life satisfaction (Nath and Pradhan, 2012). Furthermore, positive mindsets can give people the courage to overcome difficulties and build confidence (Van Schrojenstein Lantman et al., 2017). Meanwhile, wellbeing is also related to social support (Brannan et al., 2013). The higher social support is related to higher wellbeing (Schnittker, 2008). The quality of emotions an individual experiences in the daily life is one component of their wellbeing, which determines the level of life satisfaction (Diener and Chan, 2011). In addition to these, there are many factors that may affect the degree of the life satisfaction. Owing to the fact that life satisfaction is closely related to individual development, it is necessary to gain in depth knowledge on what other factors can influence life satisfaction. It is worth investigating whether or not there are links among the parent-child or peer alienation, self-concept clarity, mental resilience and life satisfaction.

### The relation between parent and child or peer alienation and life satisfaction

A study showed that the developmental direction in middle and high scholars were co-determined by the individual and the environment (Bird and Markle, 2012). As adolescence grow and mature, the external environment is more and more crucial for the developments of the adolescence. Several studies have also shown that the support from the neighbor, school, family, and peer friendship was necessary for a positive development and the sense of happiness in the early youth (Benson and Scales, 2009; Li et al., 2010). As slightly more remote relationships such as neighborhood relationships can effect adolescent developments (Brooks-Gunn et al., 1993), family support and peer friendship have more profound effects and serve protective and stimulative function for the growth of the adolescents, prompting to take shape a positive psychology and promoting life satisfaction (Masten, 2001). Adolescents’ relationships with parents and peers are crucial for their development (Chang et al., 2003). Research showed that the positive interpersonal relationship was related to the better self-regulating abilities and less criminal acts (Leung and Leung, 1992). Conversely, if an adolescent encounters parent-child alienation or peer alienation, it may have bad effects on the development, such as the mental resilience, of the adolescent (Dmitrieva et al., 2004). Previous study examining the relationship between family cohesion and life satisfaction in adolescents found that family cohesion could positively effect life satisfaction, and in turn positively effect individual self-esteem and negatively effect the tendency for depression (Lin and Yi, 2019). In addition, there was a study that showed that the relationship between parents might have an effect on the mental health development of adolescents (Leung and Leung, 1992). Thus, family relationship is significant for an adolescent’s wellbeing. Study found that if parent-child alienation or peer alienation were to take place, it would have a negative influence on men’s mental health (Sher, 2015). According to Trzcinski and Holst (2008) study, equal communications between mothers and children was related to life satisfaction. A study sampling adolescents from American and Spain found that parents support was positively correlated with life satisfaction (Stroebe et al., 2008). Relationships outside the family are more and more important as people age (Eccles, 2004), and parents involvement is a key factor for the adolescents’ scholastic achievement (Kim, 1998; Fan, 2001). The past research revealed that the high levels of school connection was of a protective strength for an adolescent, which was positively correlated with self-esteem, the adaption in the school, scholastic achievement, and study engagement (Whitlock, 2006).

Accordingly, Hypothesis 1 is proposed: parent-child or peer alienation and life satisfaction are negatively correlated.

### The mediating role of mental resilience in the relationship between parent and child or peer alienation and life satisfaction

Mental resilience is important for high-quality life and health (Van Schrojenstein Lantman et al., 2017). It represents the ability to recover from the extreme harm (Van Schrojenstein Lantman et al., 2017). The mental resilience is also related to mental heath (Hu et al., 2015) and is one of the factors of assessing the mental health of an individual (Taylor and Carr, 2018). When an individual is exposed to adverse growing environment, mental resilience can bring him or her positiveness (Van Schrojenstein Lantman et al., 2017). Researchers have a common understanding that mental resilience has two principle elements, including the ability to face adverse situation and the ability to cope successfully. The mental ability to face and overcome setbacks and challenges is a common factor used to evaluate mental resilience (Friedli and World Health Organization [WHO], 2009). Family cohesion can also influence the physical trauma and metal health, which keep in close touch with the mental resilience (Uruk et al., 2007). Studies have demonstrated that parent and peer attachment is positively related to mental resilience (Svanberg, 1998). The better the family relationship, the better the attachment type, thus, reducing alienation between families (Sandhu and Tung, 2004). In consequence, mental resilience is also an necessary factor worthy of attention during the progress of the adolescents’ growth and development. Previous literatures on the outlooks on life revealed that possessing a positive mentality was beneficial for possessing positive emotions and wellbeing (Kumpfer, 2002). People with good mental resilience have the ability to overcome difficulties and breakthrough more easily, thus possibly results in having higher life satisfaction (Kong et al., 2015). Psychological flexibility is a positive factor for mental health and life satisfaction (Moore and Lucas, 2020). Meanwhile, a research showed that building resilience could build positive emotion and in turn improve life satisfaction (Cohn et al., 2009).

Accordingly, Hypothesis 2 is proposed: mental resilience serves as a mediating role in the relationship between parent and child or peer alienation and life satisfaction.

### The mediating role of self-concept clarity in the relationship between parent and child or peer alienation and life satisfaction

Self-concept clarity refers to the cognitive evaluation of oneself’s abilities and weaknesses (Lewandowski et al., 2009). When the real self is inconsistent with the ideal self, the individual’s wellbeing maybe reduced (Nelson, 1984). According to previous studies, family relationship was seemed to be related with both self-concept clarity (Xiang et al., 2020) and life satisfaction (Chang et al., 2003). There is a relationship among high levels of parent-child interaction, environment the high self-concept clarity as well as life satisfaction (Nelson, 1984). Chen’s research on senior primary school students found that family cohesion was related to the formation of self-concept. Children in a better family cohesion environment will have a clearer self-concept compared with children with a poor family cohesion environment (Xiang et al., 2020). Different attachment typestyles are also related to different self-concept clarity. Safe-attached individuals can influence self-concept clarity through the mediating variable of self-esteem. Individuals with secure attachment style have higher levels of self-esteem and thus higher self-concept clarity. Individuals with anxiety and avoidance attachment style tendencies are negatively associated with self-certainty and self-concept clarity (Wu, 2009). Moreover, study by Streamer and Seery (2015) Seery found that family relationship and self-esteem might influence self-concept clarity in the early growth of the adolescence conjunctly and severally, and might have a great effect on their mentality constantly. The self-concept clarity is also relevant to self-representation, which is a predictor of life satisfaction (Chang et al., 2003). In the frame of multidimensional concept, depending on how relevant a specific domain of self-concept is to a developmental period, it might similarly or differently affect individuals’ subjective life satisfaction (Chang et al., 2003). According to the research field of the specific period of development, the self-concept of various fields may be influenced by life satisfaction. The self-concept of specific domain with life satisfaction may be enhanced or reduced in different periods (Chang et al., 2003). Meanwhile, self-concept clarity is also related to the mental resilience (Bigler et al., 2001). According to the previous study, having higher self-concept clarity was related to having higher self-esteem (Chui and Wong, 2016) and more positive emotions (Slotter and Walsh, 2017), which accounted for clearer self-concept that might led to happiness and higher life satisfaction. However, an individual with unclear self-concept may become lonely and dispirited and further influence life satisfaction (Watkins, 2008; Bastian et al., 2012). If an individual feel sorrowful, he or she may experience the sadness and reflect on why they feel sorrowful, their life satisfaction maybe negatively influenced (Watkins, 2008; Bastian et al., 2012).

Accordingly, Hypothesis 3 is proposed: self-concept clarity serves a mediating role in the relationship between parent and child or peer alienation and life satisfaction.

### The association between parent and child or peer alienation, self-concept clarity, mental resilience, and life satisfaction as well as the internal mechanisms of the association

Self-concept clarity is also linked to mental resilience. Self-concept clarity can affect psychological adjustment (Parise et al., 2019). When the self-clear concept clarity is strong, the level of psychological adjustment is higher, thus, having a higher mental resilience potentially (Bigler et al., 2001). According to this result, we propose that there is a relationship between mental resilience and life satisfaction. More specifically, the higher mental resilience may be associated with higher self-concept clarity.

Accordingly, Hypothesis 4 is proposed: the specific association between parent and child or peer alienation, self-concept clarity, mental resilience and life satisfaction as well as the internal mechanisms of the association.

Past studies have demonstrated that there was a relation among the parent-child or peer relationship (Chang et al., 2003), self-concept clarity (Chang et al., 2003), mental resilience (Kong et al., 2015) and life satisfaction, but how they connect concretely and what inner mechanism of their connections remained unclear and worth investigating. The current research sets forth emphatically the inner links of the several factors and knock on effect. Meanwhile, the current research addresses several important issue, including the relationship between three types of alienation and life satisfaction and the potential role of mental resilience and self-concept clarity. The questions have not been raised before. Therefore, we build three links which are mother/father/peers alienation–mental resilience–self-concept–life satisfaction in the research.

## Materials and methods

### Participants and procedure

We recruited randomly 1,565 adolescents from six middle schools in Chongqing, China in 2021. The subjects were asked to complete four different types of questionnaires on one paper within the specified time. Questionnaires not completed within the specified time and incomplete responses were excluded. Finally, 1,347 papers were collected after excluding unqualified questionnaires (incomplete questionnaires and overdue questionnaires). The sample consisted of 62.4% female participants (*n* = 841) and 37.6% male participants (*n* = 506) aged from 11 to 17 years old (Mean = 14.54, SD = 1.21). All subjects had normal visual acuity or corrected visual acuity, and no mental history.

All subjects gave their informed consent for inclusion before they participated in the study. The study was conducted in accordance with the Declaration of Helsinki, and the protocol was approved by the Ethics Committee of (Project identification code). The specialized people are responsible for distributing, collecting the questionnaire, and data entry ensuring that all participants remained anonymous to the researcher for the entire duration of the study. Each person was asked to complete questionnaires assessing. The questionnaires contain Parental and Peer Alienation, Mental Resilience, Self-Concept Clarity, and Life Satisfaction. At the end of the experiment, we collected questionnaires from each participant. Participants who failed to complete the questionnaire before the end of the experiment were excluded. Considering the ethical requirements, we only used the questionnaire method for the participants. Respect and thank the participants for their contributions.

### Instruments

The Inventor of Parent and Peer Attachment (IPPA; Guarnieri et al., 2010) is an assessment commonly used to evaluate the quality of attachments with parents and peers perceived by adolescents. The IPPA contains three subscales that, respectively, test for mother attachments, father attachments and peer attachments with 12 items in each. Each subscale incorporates three dimensions including communication, trust and alienation (reverse coded). Communication assesses the personal perception parents’ and peers’ sensibility to their emotions (e.g., “I like to get my mother’s point of view on things I am concerned about”). Trust assesses the personal perception of how much parents’ and peers’ respect and understand their needs and desires (e.g., “My mother respects my feelings”). Alienation assess personal perceptions of anger, indifference and isolation regarding the attachments (e.g., “I feel angry with my peer”). The assessment is rated on a five-point Likert scale with 1 being almost or never or never and 5 being almost always and always. Higher scores indicate higher attachment quality. We only use the alienation dimension to find relationships among variables. The scale was analyzed for its validity and the KMO value were 0.759 (mother), 0.807 (father), and 0.742 (peer). The Cronbach’s alpha for father, mother, and peer attachment subscales were 0.873, 0.886, and 0.841, respectively.

The Connor-Davidson Resilience Scale (CD-RISC-10; Burns and Anstey, 2010) was used to measure the resilience of individuals. It is the 10-item version included questions like “I can adapt changes,” “coping with pressure can make me full of strength,” “I will not be discouraged by failure.” Participants answer questions according their subjective perception of how much they agree or disagree with the statement. Questions are given on a five-point scale with 0 being “not true at all” to 4 being “true nearly all the time.” The scores are summed up with higher scores indicating higher level of resilience. The scale was analyzed for its validity and the KMO value is 0.919. The alpha of CD-RISC-10 was 0.83.

The Self-concept Clarity Scale (SCCS; Campbell et al., 1996) is a 12 itemed questionnaire often used to assess self-view. Questions include “My belief about myself often conflict with one another,” and “I spend a lot of time wondering about what kind of people I really am.”. Item 6 “I seldom experience conflict between the different aspects of my personality” and item 11 “In general, I have a clear sense of who I am and what I am” are reverse coded. Responses are given on a Likert scale of 1 to 7 with 1 being complete disagreement and 7 being complete agreement. Higher scores indicate higher self-concept clarity. The scale was analyzed for its validity and the KMO value was 0.905. The cronbach’s alpha for the SCCS was 0.85.

The Satisfaction with Life Scale (SWLS; Diener et al., 1985) is a self-report instrument used to assess the cognitive judgments of a individual’s life satisfaction. The scale contains five items and is rated on a Likert scale of 1 to 7 with 1 being strongly disagree and 7 being strongly agree. The scale was analyzed for its validity and the KMO value was 0.820. The Cronbach’s alpha for the SWLS was 0.781.

### Analytical method

Descriptive statistical analysis and regression analysis were conducted to test the associations among the variables. Next, we conducted three mediation models of father alienation, mother alienation and peer alienation on life satisfaction through mental resilience and self-concept clarity. At the same time, we also conducted two mechanism of action model including parent-child or peer alienation—mental resilience—life satisfaction and parent-child or peers alienation—self-concept clarity—life satisfaction. Finally, we tested for an bivariate: effect of parent-child or peer alienation and life satisfaction.

## Results

### Common method deviation test

According to the recommendations of Tang Dandan and Wen Zhonglin, this study used the Harman univariate test to test the common method bias of parent-child or peer alienation, mental resilience, self-concept and life satisfaction to examine the results of unrotated factor analysis (Dandan and Zhonglin, 2020). In total, 6 factors with eigenvalues greater than 1 were extracted, and the variations explained by the first common factor were 33.74% (mother), 33.80% (father), 33.70% (peer), less than the 40% critical criterion. Therefore, no common method bias was evident in the data used in this study.

### Basic descriptive analysis

The mean, standard deviation of each variable were shown in Table 1. For alienation, The average scores of mother alienation, father alienation, peers alienation were as follows: 13.29, 13.81, 11.95, ranged from 11.95 to 48.89. Besides, the total scores of alienation were 30 points. The standard deviation of three different types of alienation: mother alienation, father alienation and peer alienation were 4.18, 4.64, 3.20. For mental resilience, the average score of it is 23.02 and the total score was 40 points, while standard deviation was 6.54. For self-concept clarity, the average score, the total score and standard deviation were 48.89, 84, 12.14. For life satisfaction, The total score of the scale was 35 points, while the average value of the subjects we measured is 19.06 and the standard deviation was 5.46.

**TABLE 1:** Basic descriptive analysis.

Variables	Average scores	Total scores	Standard deviation
Mother alienation	13.29	30	4.18
Father alienation	13.81	30	4.64
Peer alienation	11.95	30	3.20
Mental resilience	23.02	40	6.54
Self-concept clarity	48.89	84	12.14
Life satisfaction	19.06	35	5.46

### Correlation analysis

We mainly used Pearson correlation coefficient to test the correlation of each variable, and the results were shown in Table 2. In the link of mother alienation–mental resilience–self-concept clarity–life satisfaction, mother alienation was negatively correlated with mental resilience (*r* = −0.226, *p* < 0.01), self-concept clarity (*r* = −0.317, *p* < 0.01), and life satisfaction (*r* = −0.320, *p* < 0.01). Mental resilience was positively correlated with self-concept clarity (*r* = 0.397, *p* < 0.01) and life satisfaction (*r* = 0.381, *p* < 0.01). Self-concept clarity was also positively correlated with life satisfaction (*r* = 0.366, *p* < 0.01). In the link of father alienation–mental resilience–self-concept clarity–life satisfaction, father alienation was negatively correlated with mental resilience (*r* = −0.242, *p* < 0.01), self-concept clarity (*r* = −0.306, *p* < 0.01), and life satisfaction (*r* = −0.303, *p* < 0.01). Mental resilience was positively correlated with self-concept clarity (*r* = 0.397, *p* < 0.01) and life satisfaction (*r* = 0.381, *p* < 0.01). Self-concept clarity was also positively correlated with life satisfaction (*r* = 0.366, *p* < 0.01). In the link of peers alienation–mental resilience–self-concept clarity–life satisfaction, mother alienation was negatively correlated with mental resilience (*r* = −0.241, *p* < 0.01), self-concept clarity (*r* = −0.331, *p* < 0.01) and life satisfaction (*r* = −0.273, *p* < 0.01). Mental resilience was positively correlated with self-concept clarity (*r* = 0.397, *p* < 0.01) and life satisfaction (*r* = 0.381, *p* < 0.01). Self-concept clarity was also positively correlated with life satisfaction (*r* = 0.366, *p* < 0.01).

**TABLE 2:** The correlation analysis among variables.

Variable name		An			MR			SCC		
		a	b	c	a	b	c	a	b	c
An	a	1			1			1		
	b		1			1			1	
	c			1			1			1
MR	a	−0.226***								
	B		−0.242***							
	c			−0.241***						
SCC	a	−0.317***			0.397***					
	b		−0306***			0.397***				
	c			−0.331***			0.397***			
LS	a	−0.320***			0.381***			0.366***		
	b		−0.303***			0.381***			0.366***	
	c			−0.273***			0.381***			0.366***

a, mother; b, father; c, peer; An, alienation; MR, mental resilience; SCC, self-concept clarity; LS, life satisfaction.

***p < 0.001.

The results showed that there were significant correlation among all variables. Meanwhile, except that alienation was negatively correlated with other variables, other variables were positively correlated.

### Regression analysis

After getting rid of the irrelevant variables that were measured in the questionnaire including self-esteem, perception and so on, we built three mediator models to elucidate the mediating role of self-concept clarity and mental resilience on the relationship between parent and child or peer alienation and life satisfaction, as shown in the Table 3.

**TABLE 3:** The regression analysis among variables.

Outcome variables	Predictive variables	*R*	*R* ^2^	F	β	*t*
Life satisfaction		0.329	0.108	54.357***		
	Mother-child alienation				−0.430***	−12.668
	Gender				−0.458	−1.574
	Age				−0.431	−2.342
Mental resilience		0.281	0.079	38.384***		
	Mother-child alienation				−0.363***	−8.787
	Gender				−2.234***	−6.306
	Age				−0.159	−0.708
Self-concept clarity		0.462	0.213	90.956***		
	Mother-child alienation				−0.711***	−9.758
	Mental resilience				0.630***	13.461
	Gender				−0.300	−0.483
	Age				−0.577	−1.500
Life satisfaction		0.487	0.237	83.454***		
	Mother-child alienation				−0.267***	−8.000
	Mental resilience				0.215***	9.747
	Self-concept clarity				0.090	7.418
	Gender				0.176	0.645
	Age				−0.336	−1.972
Outcome variables	Predictive variables	*R*	*R* ^2^	F	β	*t*
Life satisfaction		0.308	0.095	46.788***		
	Father-child alienation				−0.360***	−11.739
	Gender				−0.192	−0.655
	Age				−0.331	−1.791
Mental resilience		0.284	0.081	39.341***		
	Father-child alienation				−0.331***	−8.945
	Gender				−1.995***	−5.626
	Age				−0.088	−0.395
Self-concept clarity		0.453	0.205	86.537***		
	Father-child alienation				−0.590***	−8.962
	Mental resilience				0.637***	13.514
	Gender				0.153	0.246
	Age				−0.408	−1.060
Life satisfaction		0.48	0.23	80.0749***		
	Father-child alienation				−0.213***	−7.105
	Mental resilience				0.216***	9.710
	Self-concept clarity				0.094	7.792
	Gender				0.344	1.253
	Age				−0.268	−1.571
Outcome variables	Predictive variables	*R*	*R* ^2^	F	β	*t*
Life satisfaction		0.275	0.076	36.734***		
	Peer alienation				−0.465***	−10.377
	Gender				−0.356	−1.201
	Age				−0.096	−0.515
Mental resilience		0.288	0.083	39.310***		
	Peer alienation				−0.488***	−9.134
	Gender				−2.140***	−6.053
	Age				0.134	0.604
Self-concept clarity		0.465	0.217	92.729***		
	Peer alienation				−0.951***	−10.059
	Mental resilience				0.623***	13.302
	Gender				−0.129	−0.210
	Age				−0.003	−0.008
Life satisfaction		0.466	0.217	74.519***		
	Peer alienation				−0.235***	−5.318
	Mental resilience				0.222***	9.898
	Self-concept clarity				0.097	7.928
	Gender				0.261	0.944
	Age				−0.133	−0.778

***p < 0.001.

### Chain mediated effect test

**FIGURE 1:**
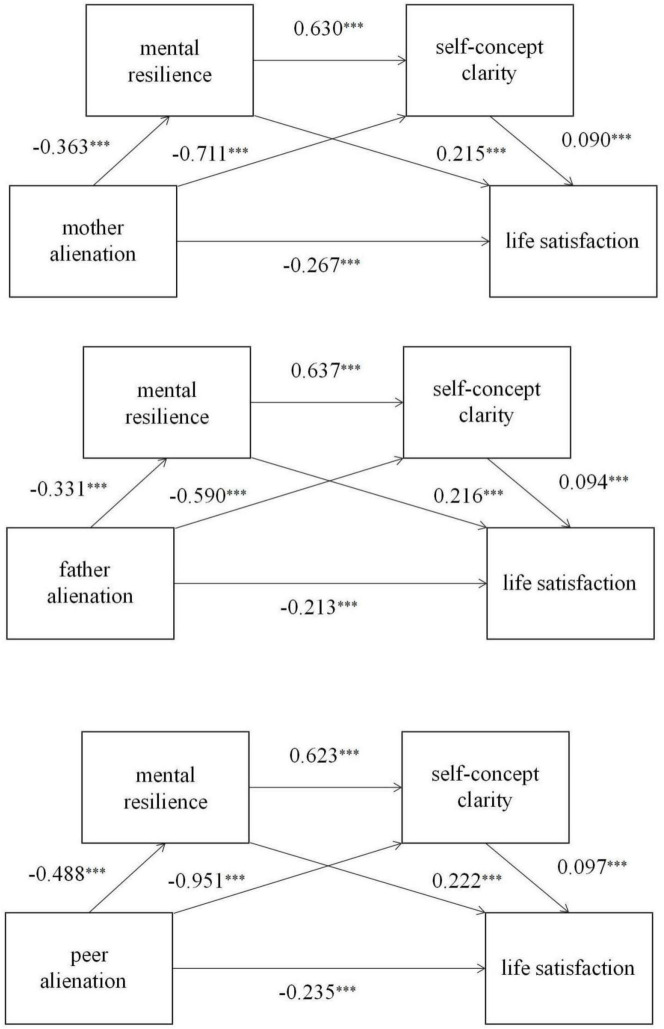
Chain intermediary model ***p < 0.001.

We used model 6 in SPSS 26 plug-in process provided by Hayes (2012). Took parent-child alienation and peer alienation as the independent variable, life satisfaction as the dependent variable, mental resilience and self-concept clarity as the chain intermediary variable, and gender as well as grade as the control variables to create the chain intermediary model, as shown in Figure 1. The whole regression equation is significant, as shown in Table 4. Bootstrap sampling method is used to test the mediating effect. The indirect effect of parental and peers alienation—mental resilience—life satisfaction was effect = −0.075 (mother), 95% CI = (−0.105, −0.053), effect = −0.072 (father), 95% CI = (−0.097, −0.049), effect = −0.108 (peer), 95% CI = (−0.148, −0.073). The indirect effect of parent-child or peer alienation—self-concept—life satisfaction was effect = −0.063 (mother), 95% CI = (−0.088, −0.042), effect = −0.056 (father), 95% CI = (−0.077, −0.037), effect = −0.092 (peer), 95% CI = (−0.126, −0.062). The indirect effect of parental and peers alienation—mental resilience—self-concept–life satisfaction was effect = −0.020 (mother), 95% CI = (−0.030, −0.012), effect = −0.021 (father), 95% CI = (−0.029, −0.012), effect = −0.300 (peer), 95% CI = (−0.043, −0.019). The sum of all indirect effects were effect = −0.162 (mother), 95% CI = (−0.198, −0.126]), effect = −0.147 (father), 95% CI = (−0.181, −0.115), effect = −0.23 (peer), 95% CI = (−0.280, −0.184). Therefore, the chain mediating effect of mental resilience, self-concept clarity in alienation and life satisfaction was established. The study found a chain of mediation model among parent-child or peer alienation, mental resilience, self-concept clarity and life satisfaction. The mediation model we did not address in our study.

**TABLE 4:** Total effect model.

	R	R-sq	MSE	F	Df1	Df2	*P*
Mother	0.329	0.108	26.633	54.357	3.000	1343.000	0.000
Father	0.308	0.095	27.040	46.788	3.000	1343.000	0.000
Peers	0.275	0.076	27.601	36.734	3.000	1343.000	0.000

## Discussion

According to the data collected, parent-child (Perrone et al., 2007; Oberle et al., 2011) or peer alienation (Oberle et al., 2011), self-concept clarity (Leung and Leung, 1992; Chang et al., 2003) and mental resilience (Fergusson et al., 2015) have an impact on life satisfaction, which is in accordance with the previous researches. The current results reveal that family environment has a direct influence on life satisfaction, which confirms previous research results (Povedano-Diaz et al., 2020). Similarly, school environment is important to life satisfaction (McCabe et al., 2011), which aligns with our results. If students are wrapped in the atmosphere of warmth, teacher support, cares and so on, it is possible to increase the quality of life subtly (Suldo et al., 2006). Additionally, low quality of parent-child or peer attachments may be accompanied with self-doubt and aggressivity (Rode et al., 2005), thus decreasing the life satisfaction. Previous research substantiated the mediating roles of self-esteem and adjustment between parental attachment and alienation from school (Kocayörük and Şimşek, 2016).

Alienation may affect children’s mental health (Sher, 2015) and cause some psychological problems, including the sense of alienation with parents and friends. Stronger alienation with less emotional communication may negatively effect life satisfaction (Moore and Lucas, 2020).

Individuals who are estranged from their parents and peers lack the opportunity to communicate their feelings and thoughts with the people around them. It is difficult to get enlightenment and help from others. In the process of growing up, it is difficult to view and evaluate themselves correctly and objectively, and eventually may not be able to form a better self-concept clarity (Tarquin and Cook-Cottone, 2008). Individuals with poor self-concept clarity also have poor life satisfaction (Chang et al., 2003). The mediating role of self-concept clarity on attachment–life satisfaction is demonstrated in the past study (Chang et al., 2003).

The result indicated that the degrees of connections with life satisfaction among these variables were different separately. For the parent-child or peer alienation, the influence of mother-child and father-child alienation on an individual’s life satisfaction was a slightly more than peer alienation. Thus, the family alienation compared with peer alienation may have a greater impact on the adolescence. Prior research found that in fifth to seventh graders, satisfaction with family was more strongly associated with the total satisfaction than satisfaction with friends (Huebner, 1991). Our study extended the participants to the high scholars and found similar results. In the current finding, mental resilience and self-concept clarity had similar mediating effects on life satisfaction, which has not been discussed in previous research.

A study found that individuals who had a sense of alienation with their parents and peers failed to have good communication with the surrounding people, separated from the surrounding environment, decreased psychological endurance and mental resilience (Sher, 2015). After reduced mental resilience, self-judgment and perception may change, which may affect self-concept clarity (Willis and Burnett, 2016).

Meanwhile, a previous research showed that mental disorder could influence life satisfaction, and life satisfaction could also influence mental disorder (Fergusson et al., 2015). The relationship remains the same when comparing between two genders. Due to the fact that a study have shown no gender difference regarding the relationships, the current study did not test for gender separately (Povedano-Diaz et al., 2020).

In addition, self-concept clarity was also a protective–mediator in the current study, which were consistent with previous research that confirmed that children growing in the warm and happy environment got a higher self-concept clarity in later stages of growth (Streamer and Seery, 2015).

Finally, there was a link of parent-child or peer alienation—mental resilience—self-concept clarity—life satisfaction. According to previous results, it was self-concept clarity and hope as two mediating variables that had a great importance in the link of family cohesion and wellbeing (Xiang et al., 2020). Our results were related with previous results. Moreover, the link of parent-child or peer alienation—mental resilience—self-concept clarity—life satisfaction was a two-mediation model, of which, mental resilience and self-concept clarity were treated as two mediators. The chain of mother alienation and life satisfaction had the largest effect, while the chain of peer alienation and life satisfaction had the least effect. Results found parent-child or peer alienation could explain life satisfaction through mediating roles of mental resilience and self-concept clarity. Parent-child or peer alienation could influence life satisfaction through the effect of mediation chain. In simpler terms, owing favorable relationship with parents and peers might endow with remarkable psychological resistance to trauma and life challenges, that was, higher levels of mental resilience. Then, having higher mental resilience might lead to clearer self-concept clarity, which might result in higher life satisfaction. It was in accordance with a previous study that mental resilience could influence self-concept clarity (Backman et al., 1963). Our results were consistent with previous results. Previous findings showed a positive association between attachment and wellbeing. However, we adopted the variable of mental resilience as part of the original chain mediation, and found a negative correlation between alienation and life satisfaction. The results added to the existing theoretical and applied knowledge.

## Conclusion

The present study found three links among parent-child or peer alienation, mental resilience, self-concept clarity and life satisfaction on the basis of previous research results. Meanwhile, finding the results that mental resilience and self-concept clarity might be the mediating variables in the influence of parent-child or peer alienation on life satisfaction. Moreover, mental resilience and self-concept clarity had moderating effect on the parent-child or peer alienation and life satisfaction, to the effect that, parent-child or peer alienation explained life satisfaction through the chain mediating effect of resilience and self-concept clarity. All in all, these finding could demonstrate family and peer relationship could influence the life satisfaction through other factors. Such a chain-based mediation model has not ever been discovered. This study explored the negative multi-influence of parent-child or peer alienation on life satisfaction and provided a new perspective for the improvement of life satisfaction of adolescents.

## Implication

In the previous study, researchers found the relationship between the parent and child or peer alienation, mental resilience, self-concept clarity, and life satisfaction severally. In our study, we studied these variables together in tandem and expanded the link including parent-child or peer alienation—mental resilience—life satisfaction, parent-child or peer alienation—self-concept clarity—life satisfaction and parent-child or peer alienation—mental resilience—self-concept clarity—life satisfaction, of which helped us have more profound understanding on the potential relationship among these variables and found underlying mechanisms of inner link. Thus, we found the chain mediation model. Additionally, whether achievement was contacted with life satisfaction or not, which was worth to studying.

## Limitations and future directions

This research had some limitations. First, personality traits were not controlled in the study. Second, the link of highlight in the current studied the relationship of parent-child or peer alienation and life satisfaction when treating mental resilience and self-concept clarity as mediations. Third, the study did not consider the relation in family. The variables of the current study could have other relationships such as the link between parent and child or peer alienation and self-concept clarity when treating mental resilience and life satisfaction as mediations remain to be studied. In the future, researchers can study the link mentioned above to find more different links among these variables and can expand the age of the participants. Meanwhile, our study only used questionnaires to obtain information about several variables, and there might have bias the results due to the subjectivity of the subjects. Therefore, in future studies, the way of collecting variable data can be expanded to make the results more accurate.

## Data availability statement

The raw data supporting the conclusions of this article will be made available by the authors, without undue reservation.

## Ethics statement

The studies involving human participants were reviewed and approved by the School of psychology, Southwest University. Written informed consent to participate in this study was provided by the participants or their legal guardian/next of kin.

## Author contributions

NC, YJ, and YP: conceptualization and investigation. YJ and YP: methodology and writing – review and editing. YP: formal analysis. YJ: resources. NC: data curation. NC and YJ: writing – original draft preparation. All authors have read and agreed to the published version of the manuscript.
